# Comparison of surface integrity, longevity of posterior tooth-colored crowns fabricated by conventional, CAD/CAM, and 3D printing techniques

**DOI:** 10.3389/fdmed.2026.1900498

**Published:** 2026-08-18

**Authors:** Tanner Hunt, Jacob Nichols, Preston Harmon, Neamat Hassan Abubakr

**Affiliations:** 1 School of Dental Medicine, University of Nevada, Las Vegas, NV, United States; 2 Biomedical Sciences Department, School of Dental Medicine, University of Nevada, Las Vegas, NV, United States

**Keywords:** 3D printing ceramic, CAD/CAM, posterior crowns, surface roughness, survival rate

## Abstract

**Introduction:**

This *in vitro* study evaluated internal and marginal surface roughness and simulated thermocycling survival of posterior tooth-colored crowns fabricated using conventional laboratory, CAD/CAM milling, and digital light processing 3D printing workflows.

**Methods:**

The roughness analysis included 46 crowns allocated to six groups: lab-made crowns (*n* = 6), IPS e.max CAD (*n* = 8), IPS Empress CAD (*n* = 8), Lava Ultimate (*n* = 8), IPS e.max ZirCAD (*n* = 8), and 3 D SprintRay Ceramic (*n* = 8). Internal and marginal arithmetic mean roughness (Ra, µm) were measured with a 3D non-contact profilometer. Because residual normality and variance homogeneity assumptions were not satisfied, group differences were analyzed using Kruskal–Wallis tests followed by Holm-adjusted pairwise Mann–Whitney *U* tests.

**Results:**

Internal Ra differed significantly among groups (*H* = 25.76, df = 5, *p* < 0.001). 3D printed SprintRay Ceramic showed the highest internal Ra (29.27 ± 5.45 µm) and was significantly rougher internally than every other group after Holm correction. Marginal Ra also differed significantly among groups (*H* = 41.86, df = 5, *p* < 0.001). IPS e.max CAD showed the highest marginal Ra (26.81 ± 1.73 µm), followed by lab-made crowns (19.31 ± 6.40 µm), whereas IPS e.max ZirCAD showed the lowest marginal Ra (4.82 ± 1.03 µm).

**Conclusion:**

Within the limitation of the present study material type and fabrication workflow significantly influenced internal and marginal surface roughness of posterior tooth-colored crowns. The crowns survival rates were more closely associated with material type than fabrication method.

## 1 Introduction

Digital dentistry has markedly altered the fabrication of fixed prosthodontic restorations through the combination of intraoral scanning, computer-aided design/computer-aided manufacturing (CAD/CAM), and, more recently, additive manufacturing. Conventional laboratory procedures and subtractive CAD/CAM milling remain established methods for producing fixed restorations, particularly tooth-colored ceramic crowns. In comparison, emerging three-dimensional (3D) printing technologies are under investigation as alternative fabrication approaches because of their potential to simplify workflows, reduce laboratory processing steps, improve reproducibility, and minimize both chairside and laboratory time. However, the clinical success of fixed restorations is highly dependent on the accuracy of fit. Marginal and internal adaptations are affected by factors such as fabrication technique, digital workflow, material selection, milling or printing parameters, and cement-space design. Previous studies have shown that CAD/CAM systems can produce clinically acceptable marginal and internal fit, although considerable variation exists among systems and fabrication protocols (1, 2). Systematic reviews also indicate that marginal adaptation is affected by multiple factors, including restorative material, finish-line design, cement space, and manufacturing method (3, 4).

Marginal integrity is critical because poorly adapted margins can contribute to microleakage, cement dissolution, plaque accumulation, recurrent caries, and gingival inflammation, all of which may compromise the long-term prognosis of crowns (3). Surface roughness is another clinically relevant factor, as rough ceramic or restorative surfaces can increase plaque retention and biofilm formation and may negatively affect restoration longevity (5). From a bonding perspective, controlled internal surface roughness may enhance micromechanical interaction with resin-based luting agents. In contrast, excessive roughness or irregularity at the crown margin can create voids, interfere with seating, and increase plaque retention at the restoration–tooth interface. Thus, evaluating both the internal surface and marginal surface characteristics is essential when comparing conventional, CAD/CAM-milled, and additively manufactured fixed restorations. Recent evidence from Sanches et al. found no clear superiority of CAD/CAM crowns over heat-pressed lithium disilicate crowns in terms of marginal adaptation, while also noting substantial heterogeneity among the included studies and the need for standardized evaluation methods (4). These conclusions support continued inquiry into how different fabrication methods influence the marginal integrity and surface roughness of fixed restorations.

CAD/CAM milling is a subtractive manufacturing process that produces restorations from prefabricated ceramic or hybrid blocks (6, 7). Although this workflow provides predictable material properties and clinically acceptable accuracy, the marginal and surface characteristics of milled restorations may be influenced by fabrication and post-processing procedures, including thermal aging, sintering or crystallization, glazing, and polishing (6–8). Additive manufacturing, in contrast, produces restorations layer by layer, and its accuracy and surface characteristics are affected by the printing technology, build orientation, layer thickness, and subsequent post-processing procedures (9–11). Additive manufacturing may also reduce material waste and expand the range of applications of digital technology in dentistry (10–12).

Despite the rapid adoption of digital workflows, comparative information remains limited regarding the surface integrity and simulated durability of posterior tooth-colored crowns fabricated by conventional laboratory, CAD/CAM, and 3D printing techniques under standardized conditions. Therefore, the objective of this *in vitro* study was to compare internal and marginal surface roughness and simulated thermocycling survival of posterior tooth-colored crowns fabricated using different techniques. The null hypothesis was that material type and fabrication method would not significantly affect internal surface roughness, marginal surface roughness, or survival after thermocycling.

## 2 Materials and methods

### 2.1 Ethical approval and specimen preparation

The University of Nevada, Las Vegas Office of Research Integrity–Research Protections reviewed this project (Protocol No. UNLV-2026-347) and determined that it did not meet the federal definition of research involving human subjects under 45 CFR 46. Therefore, further Institutional Review Board review was not required. The project used extracted human teeth, and no identifiable patient information was collected, recorded, or analyzed. All specimens were handled in accordance with applicable institutional procedures.

The sample size was calculated from a previous study (13) using an alpha level of 0.05 and a statistical power of 80%. The calculated minimum sample size was 30 teeth. A total of 54 extracted posterior teeth were initially collected and assessed for eligibility. Six teeth were excluded because they did not meet the standardized preparation criteria. The remaining 48 teeth were prepared and allocated equally among six material/fabrication groups, with eight teeth assigned to each group. During crown fabrication, two specimens assigned to the conventional laboratory-fabricated group were lost during processing by the commercial dental laboratory. Consequently, six conventional laboratory-fabricated crowns and eight crowns from each of the other five groups were available for evaluation, resulting in a final sample of 46 crowns. All 46 crowns were included in the surface-roughness and thermocycling analyses.

Caries-free, recently extracted human molars with comparable mesiodistal and buccolingual dimensions were obtained from the Oral Surgery Clinic at the School of Dental Medicine, University of Nevada, Las Vegas. Teeth were disinfected in 0.5% chlorine solution, cleaned with an ultrasonic scaler to remove soft tissue remnants and calculus, sterilized by autoclaving, and stored in distilled water at room temperature until testing.

**Figure 1:**
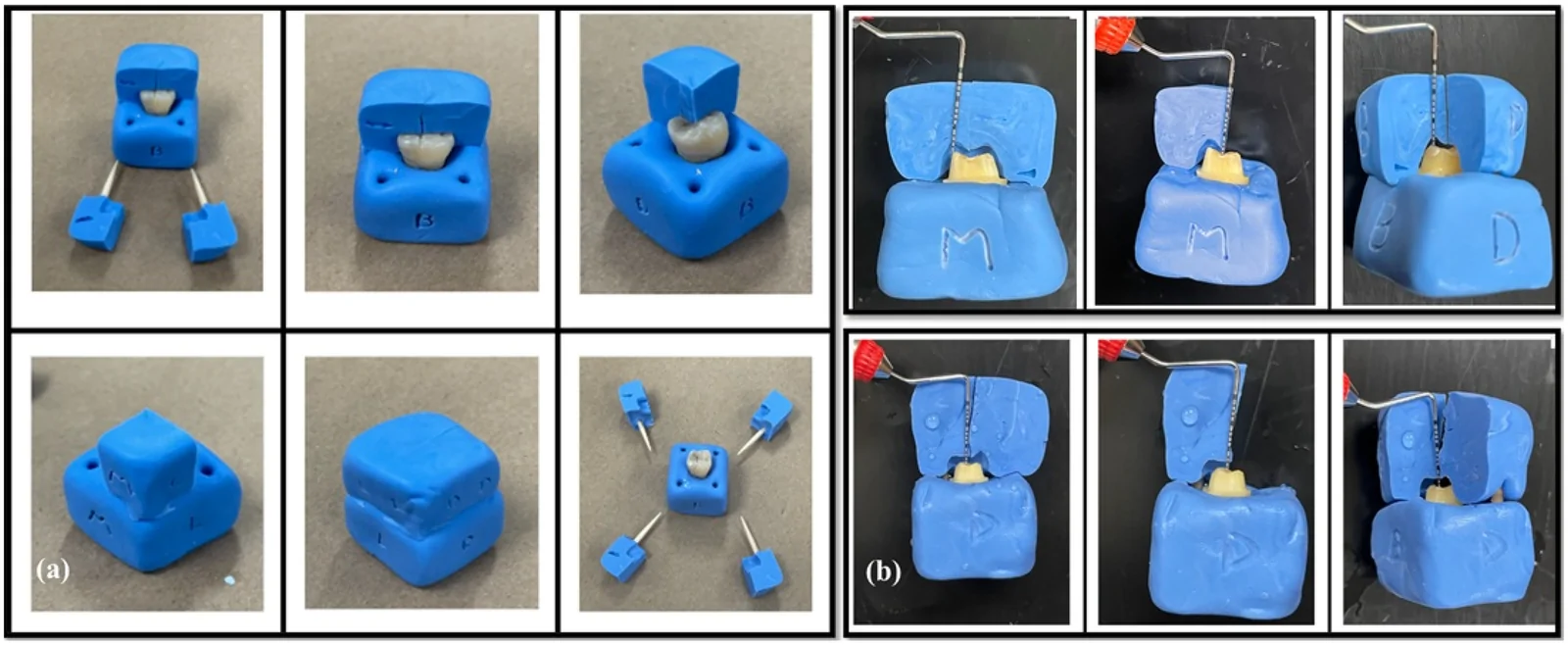
**(a)** preparation of the PVS index to standardize ceramic crown preparation. **(b)** Standardized crown preparation reduction measured with periodontal probe.

Each tooth was vertically embedded in vinyl polysiloxane (VPS; Kerr Extrude XP, Kerr Corp., Romulus, MI, USA) for stabilization. Additional VPS was used to create reduction guides. Toothpicks were placed at the corners of the VPS molds to maintain alignment of occlusal and apical impressions. Mesial, lingual, distal, and buccal reference marks were placed on each specimen. The VPS index was sectioned into quadrants to facilitate standardized three-dimensional assessment of preparation reduction (Figure 1a).

### 2.2 Crown preparation

All teeth were prepared for full-coverage posterior crowns according to manufacturer recommendations, with a minimum 1.0-mm flat shoulder margin with rounded internal line angles, 1.5-mm occlusal reduction, and 1.5-mm axial reduction. A periodontal probe and the VPS reduction guide were used to standardize the reduction before crown fabrication (Figure 1b) (8).

Preparation dimensions were standardized and verified using a calibrated periodontal probe and the sectioned VPS reduction guide. Before tooth preparation, the VPS index was fabricated to record the original tooth contour. After preparation, the sectioned VPS guide was repositioned on each tooth to compare the prepared surfaces with the original contour. The 1.0-mm shoulder margin was confirmed by placing the periodontal probe perpendicular to the finish line at the buccal, lingual, mesial, and distal surfaces. The 1.5-mm occlusal reduction was verified by measuring the space between the occlusal surface of the prepared tooth and the corresponding internal surface of the VPS guide. The 1.5-mm axial reduction was verified at the buccal, lingual, mesial, and distal surfaces using the same VPS guide and periodontal probe.

To ensure consistency, the periodontal probe was inspected before measurement, and the same measurement procedure was applied to all specimens. Reduction was checked at predetermined reference areas corresponding to the mesial, distal, buccal, lingual, and occlusal surfaces. If the intended preparation dimensions were not achieved, the preparation was adjusted and remeasured until the required reduction was confirmed. Specimens that could not be standardized according to the preparation criteria were excluded before crown fabrication. Six teeth were excluded because they did not meet the standardized preparation criteria. The remaining 48 teeth were prepared and allocated equally among six material/fabrication groups, with eight teeth assigned to each group.

### 2.3 Crown fabrication

**Figure 2:**
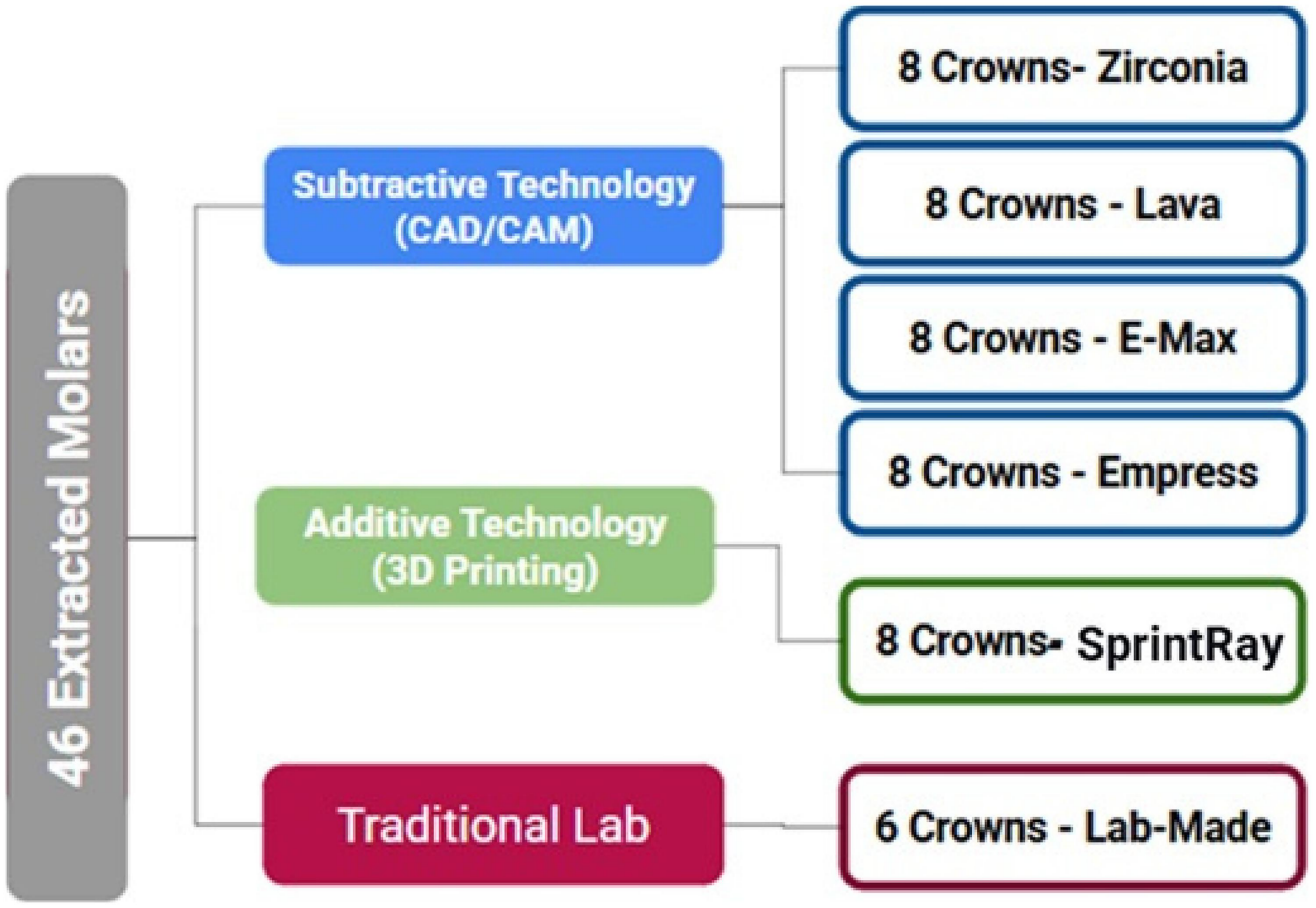
Flowchart indicating the breakdown by manufacturing technique and materials used.

Crowns were fabricated using three different manufacturing methods: CAD/CAM milling (subtractive technology), 3D printing (additive technology), and conventional laboratory fabrication. As illustrated in Figure 2, the subtractive group comprised four different material subgroups, while the additive and conventional groups each represented a single category.

#### 2.3.1 CAD/CAM milling

The CAD/CAM-milled groups included IPS e.max ZirCAD zirconia blocks (Ivoclar Vivadent, Schaan, Liechtenstein), Lava Ultimate resin nano-ceramic blocks (3M, St. Paul, MN, USA), IPS e.max CAD lithium disilicate blocks (Ivoclar Vivadent Schaan, Liechtenstein), and IPS Empress CAD leucite-reinforced glass ceramic blocks (Ivoclar Vivadent Schaan, Liechtenstein). Eight crowns were fabricated from each material.

Each prepared tooth was scanned with an Emerald intraoral scanner (Planmeca/D4D Technologies, Richardson, TX, USA). Crown designs were generated using Romexis CAD/CAM software (Planmeca/D4D Technologies, Richardson, TX, USA), and cervical margins were defined using default software parameters.

Designs were milled using a PlanMill 40S milling machine (Planmeca/D4D Technologies, Richardson, TX, USA) at the University of Nevada, Las Vegas School of Dental Medicine. Milling was performed under wet conditions using 1.0-mm and 0.6-mm diamond burs and manufacturer-specific milling strategies.

Post-processing was performed according to manufacturer recommendations. IPS e.max CAD crowns were crystallized at 850 °C for 30 min in a Programat P500 furnace (Ivoclar Vivadent, Schaan, Liechtenstein), and glazed with IPS e.max Crystall/Glaze. IPS Empress CAD crowns were glazed at 770 °C for 25 min. IPS e.max ZirCAD crowns were sintered at 1,500 °C for 2 h and then glaze-fired. Lava Ultimate crowns were finished and polished according to the manufacturer's instructions.

#### 2.3.2 3D printing

3D ceramic crowns were fabricated using SprintRay Ceramic Crown (SprintRay Inc., Los Angeles, CA, USA) with a SprintRay Pro 95 S 3D printer using digital light processing technology. For 3D ceramic SprintRay crowns, standard tessellation language files were exported to the printer. Printing was performed using manufacturer-recommended settings: 100 μm layer thickness, 405-nm ultraviolet LED light source, and approximately 30 mm/h print speed. Crowns were oriented with the occlusal surface facing and parallel to the build platform, with the long axis of the restoration approximately 90 ° to the platform and minimal support structures. 3D printed ceramic crowns were washed manually twice in more than 91% isopropyl alcohol for 1 min each, dried with compressed air, and post-cured for 6 min and 42 s in the center of the SprintRay ProCure 2 tray under 405-nm LED light. Manufacturer-recommended light polishing was performed before analysis.

#### 2.3.3 Conventional laboratory fabrication

Eight prepared teeth were assigned to the conventional laboratory-fabricated crown group and sent to a commercial dental laboratory for crown fabrication. Six completed crowns were returned and included in the study. Two specimens were lost during laboratory processing and were therefore unavailable for evaluation. Accordingly, the final conventional laboratory-fabricated group consisted of six crowns.

No specimens were lost during the fabrication of the CAD/CAM or 3D printed crowns. Consequently, the final conventional laboratory-fabricated group consisted of six crowns, whereas each of the remaining groups consisted of eight crowns.

### 2.4 Internal surface roughness measurement

**Figure 3:**
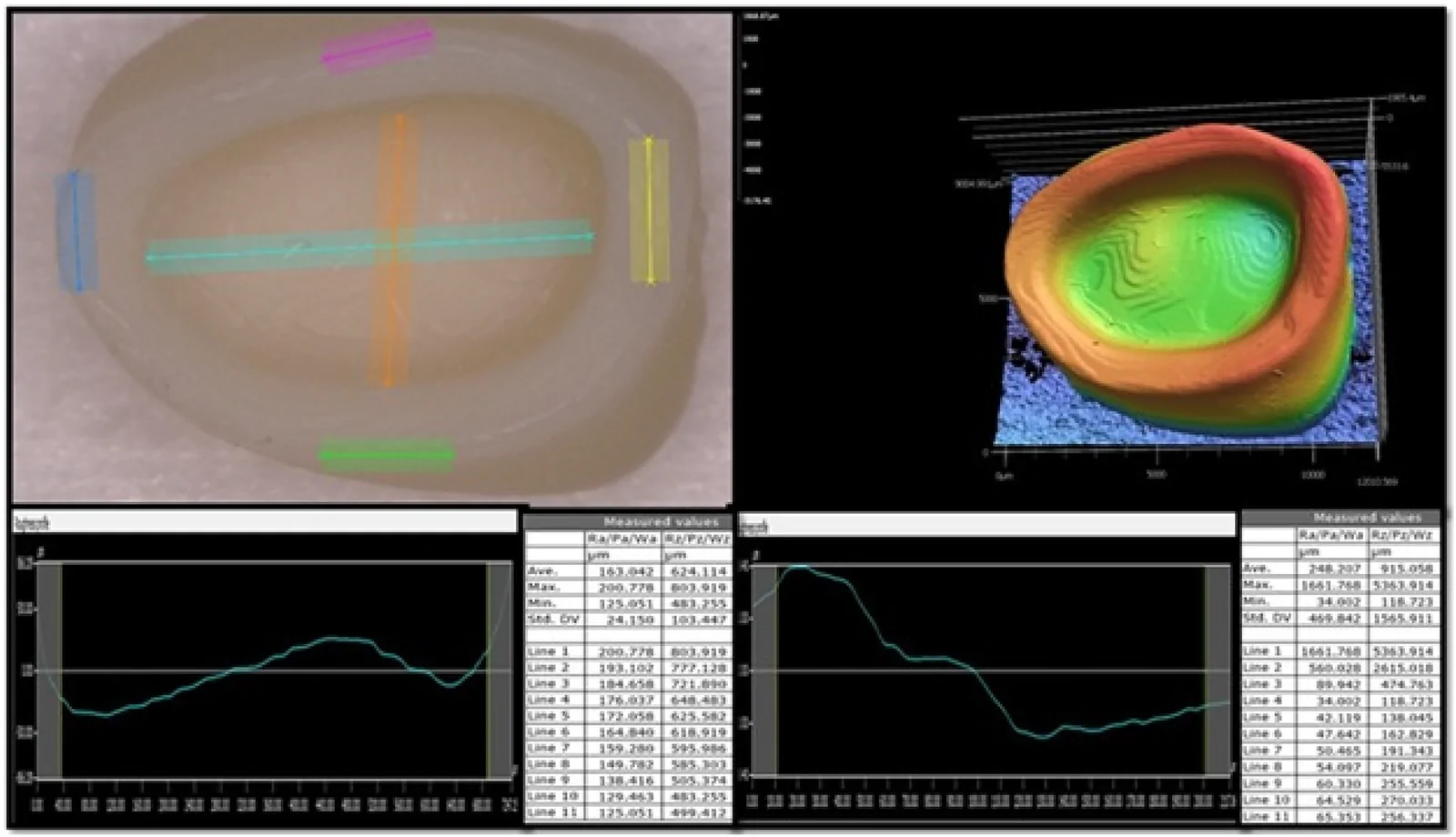
Three-dimensional non-contact profilometer analysis of internal and marginal surface roughness measurements. Representative line scans and surface topography were used to calculate arithmetic mean roughness values.

The internal surface of each crown was evaluated using a 3D non-contact profilometer (VR-3100; Keyence, Osaka, Japan). Two measurement sites were selected at the mesiodistal and buccolingual line-angle regions of the internal surface. Surface topography was analyzed using the manufacturer's software (Figure 3). Although maximum, minimum, average roughness, and peak-to-valley values were generated, arithmetic mean roughness (Ra, μm) was selected as the primary outcome to allow comparison with previous work (13).

The internal surface measurement sites were selected at standardized mesiodistal and buccolingual regions of each crown because these areas provided reproducible reference locations and represented clinically relevant surfaces that may influence crown seating and internal adaptation. These regions may also reflect differences produced by fabrication methods, including CAD/CAM milling, 3D printing, and conventional laboratory processing. For standardization, measurement sites were identified using the previously marked mesial, distal, buccal, and lingual orientations, and the measurement lines were positioned consistently in corresponding internal regions for all crowns.

### 2.5 Marginal surface roughness measurement

Marginal surface roughness was assessed from the same scans. Two points were placed along each of the four cervical margins, and surface profiling between these points was performed using Keyence software (Figure 3). Average Ra was used as the primary marginal roughness outcome. Before data collection, measurement locations and evaluation procedures were calibrated among investigators to improve consistency across samples.

Marginal surface roughness was assessed using standardized line-profile measurements along the cervical margin. Measurement points were selected using the mesial, distal, buccal, and lingual reference orientations placed on each specimen. The same measurement protocol was applied to all crowns to maintain consistency in scan location, line placement, and Ra recording. This approach allowed reproducible profiling of the marginal surface while reducing variability related to crown morphology.

All scans were performed using the same profilometer settings and software parameters. Crowns were oriented consistently during imaging to ensure that the internal and marginal surfaces were captured under comparable conditions. Before data collection, the measurement protocol was reviewed by the investigators, and representative specimens were measured jointly to ensure agreement regarding point selection, scan orientation, and Ra recording.

### 2.6 Examiner reliability

To assess measurement reliability, a subset of specimens was remeasured under identical conditions after a two week interval. Intra-examiner reliability was evaluated using the intraclass correlation coefficient (ICC), as Ra values represent continuous measurements. The intra-examiner ICC was 0.81, indicating good reliability and confirming acceptable consistency in measurement-site selection and surface roughness assessment.

### 2.7 Crown cementation and thermocycling

Crowns included in the thermocycling survival analysis were cemented with Panavia V5 adhesive resin cement (Kuraray Noritake Dental Inc., Tokyo, Japan) according to the manufacturer's instructions. Thermal aging was performed using an SD Mechatronik thermocycler (SD Mechatronik GmbH, Feldkirchen-Westerham, Germany) with water baths maintained at 5 °C and 55 °C. Each cycle consisted of 20 s of immersion in each bath. Based on previous literature, 10,000 thermocycles are considered equivalent to approximately one clinical year (14). Crown retention and survival were evaluated after 10,000, 30,000, 50,000, 70,000, and 100,000 thermocycles, corresponding to approximately 1, 3, 5, 7, and 10 simulated clinical years, respectively. At each interval, specimens were removed from the thermocycler, rinsed, dried, and examined independently under standardized conditions by two calibrated examiners blinded to specimen group allocation and fabrication records. Inter-examiner reliability was demonstrated by a calibration coefficient of 0.81. Failure was defined as complete or partial debonding, visible crown fracture, crack formation or propagation, chipping, or any loss of structural integrity. Crowns without evidence of any predefined failure were classified as surviving the corresponding thermocycling interval.

### 2.8 Statistical analysis

Internal and marginal Ra values were summarized using the mean and standard deviation, as well as the median and range. Normality was assessed using the Shapiro–Wilk test, and homogeneity of variance was evaluated using Levene's test. Because both outcomes violated the assumptions of normality and homogeneity of variance, nonparametric statistical analyses were performed. Differences among the six material and fabrication groups were evaluated using the Kruskal–Wallis test. When the omnibus test indicated a statistically significant difference, *post hoc* pairwise comparisons were performed using Mann–Whitney *U* tests, with *p*-values adjusted using the Holm procedure to account for multiple comparisons. Epsilon-squared (*ε*^2^) was calculated as the effect-size measure for each Kruskal–Wallis omnibus comparison to quantify the magnitude of the overall between-group differences. Effect sizes were interpreted as small, moderate, or large according to established thresholds. The omnibus test results, effect sizes, effect-size interpretations, and principal Holm-adjusted pairwise findings are presented in Table 1. The level of statistical significance was set at *α* = 0.05.

**Table 1:** Omnibus statistical comparisons, effect sizes, and significant pairwise findings for internal and marginal surface roughness.

Outcome	Assumption checks	Omnibus statistical test	Effect size, *ε*^2^	Effect-size interpretation	Holm-adjusted pairwise findings
Internal Ra	Shapiro–Wilk, *p* < 0.001; Levene test, *p* = 0.0005	Kruskal–Wallis *H* = 25.76, df = 5, *p* < 0.001	0.52	Large	SprintRay Ceramic Crown demonstrated significantly greater internal Ra than all other groups. The adjusted *p*-value was 0.0023 for comparisons with IPS e.max CAD, IPS Empress CAD, Lava Ultimate, and IPS e.max ZirCAD, and 0.0073 for the comparison with conventional laboratory-fabricated crowns. No other pairwise comparison remained statistically significant after Holm correction.
Marginal Ra	Shapiro–Wilk, *p* < 0.001; Levene test, *p* < 0.001	Kruskal–Wallis *H* = 41.86, df = 5, *p* < 0.001	0.92	Very large	All 15 pairwise comparisons remained statistically significant after Holm correction (adjusted *p* ≤ 0.0426). IPS e.max CAD demonstrated the highest marginal Ra and was significantly rougher than all other groups. IPS e.max ZirCAD demonstrated the lowest marginal Ra and was significantly smoother than all other groups.

Thermocycling survival was summarized descriptively as the number and percentage of crowns retained after each simulated aging interval. Because specimen numbers were small and unequal across groups, and because cyclic mechanical loading was not performed, survival findings were interpreted as exploratory *in vitro* thermocycling outcomes rather than estimates of clinical survival.

## 3 Results

### 3.1 Descriptive roughness outcomes

The roughness analysis included 46 crowns. Mean internal and marginal Ra values are shown in Table 2. 3D Ceramic crowns showed the highest internal roughness (29.38 ± 5.87 μm), whereas lab-made crowns showed the lowest mean internal roughness values (3.84 ± 0.60 μm). IPS e.max CAD showed the highest marginal roughness (26.81 ± 1.73 μm), followed by lab-made crowns (19.31 ± 6.40 μm). IPS e.max ZirCAD showed the lowest marginal roughness (4.82 ± 1.03 μm).

**Table 2:** Descriptive statistics for internal and marginal surface roughness by material group.

Material group	Fabrication/material category	Number	Internal Ra (µm), mean ± SD	Internal Ra median (range)	Marginal Ra (µm), mean ± SD	Marginal Ra median (range)
Lab-made	Conventional laboratory	6	3.84 ± 0.60	3.90 (2.86–4.75)	19.31 ± 6.40	18.35 (13.06–29.69)
IPS e.max CAD	CAD/CAM-milled lithium disilicate	8	4.54 ± 0.57	4.45 (3.87–5.62)	26.81 ± 1.73	26.64 (24.52–29.15)
IPS Empress CAD	CAD/CAM-milled leucite-reinforced glass ceramic	8	4.46 ± 1.49	3.63 (3.29–7.30)	7.02 ± 0.76	6.99 (5.58–7.93)
Lava Ultimate	CAD/CAM-milled resin nano-ceramic	8	5.61 ± 1.82	5.17 (3.64–8.57)	9.78 ± 1.61	9.44 (7.80–12.30)
IPS e.max ZirCAD	CAD/CAM-milled zirconia	8	4.73 ± 0.95	4.33 (3.96–6.69)	4.82 ± 1.03	5.26 (3.10–5.75)
3D SprintRay Ceramic	DLP 3D printed ceramic resin	8	29.27 ± 5.45	26.92 (24.34–38.33)	8.30 ± 0.41	8.32 (7.56–8.86)

### 3.2 Statistical comparison of internal surface roughness

For internal Ra, residual normality and homogeneity of variance were not satisfied (Shapiro–Wilk *p* < 0.001; Levene *p* = 0.0005). Therefore, a Kruskal–Wallis test was used. Internal Ra differed significantly among groups (*H* = 25.76, df = 5, *p* < 0.001). 3D SprintRay Ceramic showed significantly higher internal Ra than all other groups after Holm correction (adjusted *p* = 0.0023 vs. IPS e.max CAD, IPS Empress CAD, Lava Ultimate, and IPS e.max ZirCAD; adjusted *p* = 0.0073 vs. lab-made crowns). No other internal Ra pairwise comparison remained significant after Holm correction.

### 3.3 Statistical comparison of marginal surface roughness

For marginal Ra, residual normality and homogeneity of variance were not satisfied (Shapiro–Wilk *p* < 0.001; Levene *p* < 0.001). Therefore, a Kruskal–Wallis test was used. Marginal Ra differed significantly among groups (*H* = 41.86, df = 5, *p* < 0.001). All Holm-adjusted pairwise comparisons for marginal Ra were statistically significant (adjusted *p* ≤ 0.0426) (Table 2). IPS e.max CAD had the highest marginal Ra and was significantly rougher than all other groups. Lab-made crowns were significantly rougher than IPS Empress CAD, Lava Ultimate, IPS e.max ZirCAD, and 3D SprintRay Ceramic, but significantly less rough than IPS e.max CAD. IPS e.max ZirCAD had the lowest marginal Ra and was significantly smoother than every other group.

The omnibus statistical comparisons, effect sizes, and principal *post hoc* findings are summarized in Table 1. Internal surface roughness differed significantly among the six groups (Kruskal–Wallis *H* = 25.76, df = 5, *p* < 0.001), with a large effect size (*ε*^2^ = 0.52). Marginal surface roughness also differed significantly among the groups (Kruskal–Wallis *H* = 41.86, df = 5, *p* < 0.001), with a very large effect size (*ε*^2^ = 0.92). These findings indicate substantial between-group differences in both internal and marginal surface roughness according to the evaluated material and fabrication method.

### 3.4 Thermocycling survival outcomes

**Figure 4:**
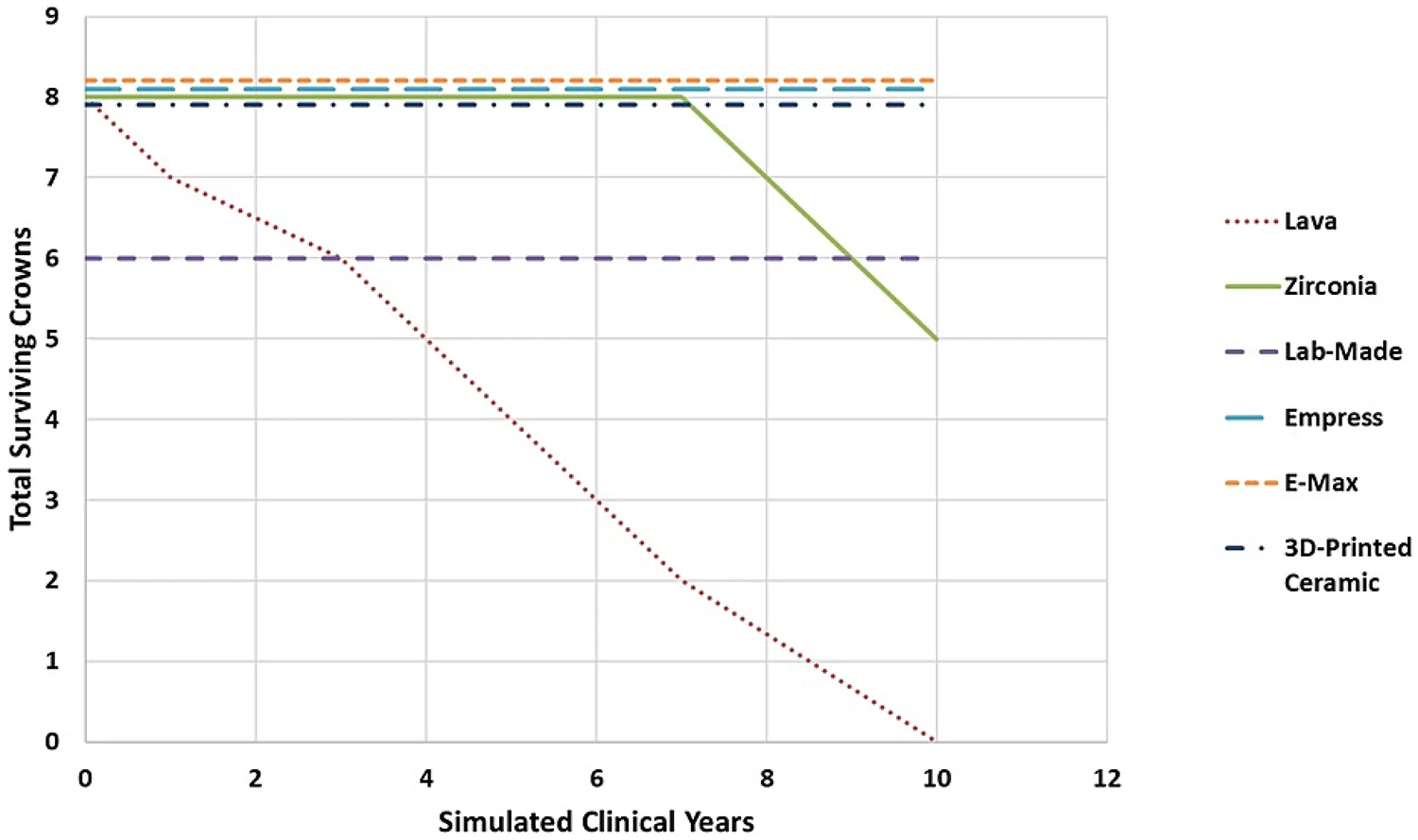
Survival of crowns through the 10 simulated clinical years.

Thermocycling survival analysis showed that 11 crowns failed during simulated aging. IPS Empress CAD, IPS e.max CAD, conventional laboratory-fabricated crowns, and 3D printed SprintRay Ceramic crowns demonstrated 100% survival throughout the simulated 10-year aging period, indicating that no failures were observed in these groups under the applied thermocycling conditions. In contrast, Lava Ultimate crowns exhibited complete failure by the end of the simulated 10-year aging period, suggesting lower resistance to thermocycling-related degradation in this group. IPS e.max ZirCAD crowns showed intermediate survival, with three of the eight crowns failing during simulated aging, corresponding to a survival rate of 62.5%. Overall, these findings indicate material-dependent differences in survival following prolonged thermocycling, with ceramic and conventional laboratory-fabricated crowns generally demonstrating higher survival than Lava Ultimate under the conditions of this study (Figure 4).

## 4 Discussion

This study compared the internal and marginal surface roughness of posterior crowns fabricated using conventional laboratory procedures, subtractive CAD/CAM milling, and DLP 3D printing. The findings demonstrated substantial differences among the evaluated groups and indicated that internal and marginal surfaces should be interpreted separately, as they serve distinct clinical functions. SprintRay Ceramic Crown exhibited the highest internal surface roughness, whereas the CAD/CAM-milled and conventional laboratory-fabricated crowns generally demonstrated lower internal roughness values. A recent systematic review similarly reported that both additive and subtractive manufacturing methods can produce clinically acceptable internal and marginal fit for fixed prosthodontic restorations (15).

This study adds to the available evidence by directly comparing the internal and marginal surface roughness of crowns fabricated using different manufacturing approaches, including subtractive CAD/CAM milling, additive 3D printing, and conventional laboratory fabrication. While previous studies have frequently focused on marginal fit, internal adaptation, or mechanical properties, the present investigation highlights the importance of evaluating internal and marginal surface roughness as separate but clinically related outcomes. This distinction is important because the internal crown surface may influence cement interaction and retention, whereas the marginal surface may influence plaque accumulation, marginal discoloration, microleakage, and periodontal response. Therefore, the findings provide clinically relevant information for selecting fabrication methods that balance bonding potential with marginal surface quality.

Thermocycling was used to expose the specimens to repeated temperature changes similar to those occurring during the consumption of hot and cold foods and beverages. ISO 11405 describes 500 cycles between 5 °C and 55 °C as a short-term aging protocol (16), whereas Gale and Darvell proposed that approximately 10,000 cycles may correspond to nearly one year of clinical thermal exposure (14). Nevertheless, thermocycling reproduces thermal stress only and does not simulate the combined effects of mastication, cyclic loading, saliva, pH changes, or biofilm activity. The thermocycling findings should therefore be interpreted as exploratory *in vitro* aging outcomes rather than estimates of long-term clinical survival.

Marginal surface roughness is clinically relevant because rough surfaces may facilitate plaque retention and bacterial adhesion, potentially increasing the risk of gingival inflammation, marginal discoloration, microleakage, and recurrent caries (5, 17–21). In the present study, IPS e.max CAD and conventional laboratory-fabricated crowns demonstrated the highest marginal Ra values, whereas IPS e.max ZirCAD demonstrated the lowest. SprintRay Ceramic Crown exhibited high internal roughness but comparatively low marginal roughness, further confirming that roughness at the internal and marginal surfaces does not necessarily follow the same pattern.

The observed differences may be explained by the distinct manufacturing and post-processing procedures used for each group. CAD/CAM-milled restorations may be affected by milling-bur diameter, bur wear, milling strategy, material hardness, crystallization or sintering procedures, and the ability of the bur to reproduce fine surface details. DLP 3D printed restorations may be influenced by layer thickness, build orientation, resin viscosity, ceramic filler content, light penetration, polymerization depth, and post-curing procedures. Conventional laboratory fabrication may additionally be influenced by technician-dependent waxing, investing, pressing, finishing, and polishing. These variables may account for the different internal and marginal surface topographies observed among groups.

Differences among lithium disilicate, leucite-reinforced glass-ceramic, resin nanoceramic, IPS e.max ZirCAD, and 3D printed ceramic-filled resin also indicate that surface integrity is influenced by both material composition and processing conditions. Previous research has shown that the microstructure and surface topography of pressed and milled lithium disilicate restorations differ and may affect their adaptation and bonding behavior (22). The present findings similarly suggest that material properties should be evaluated together with the manufacturing method rather than considered independently.

SprintRay Ceramic Crown demonstrated lower marginal roughness than several milled and conventionally fabricated groups, despite exhibiting the highest internal roughness. This finding suggests that additive manufacturing can produce substantially different surface characteristics at separate crown regions. However, the combination of a rougher internal surface and smoother marginal surface should not be described as a clinical advantage without direct evidence of improved bond strength, seating accuracy, marginal fit, or clinical performance. Aldahian et al. reported favorable fit and adaptation for 3D printed interim restorations compared with conventional and CAD/CAM-fabricated restorations, although the printed restorations also demonstrated greater internal surface roughness (23).

Vat polymerization methods used in dentistry include stereolithography and digital light processing. Stereolithography polymerizes resin point by point using a focused laser, whereas DLP projects and polymerizes an entire layer simultaneously (24, 25). These technologies differ in their exposure methods, printing speeds, and resolutions (26). Ceramic fillers are incorporated into printable resins to improve mechanical and thermal properties (27); however, filler type, size, and concentration may alter viscosity, light transmission, polymerization depth, interlayer bonding, and dimensional accuracy (28).

DLP was selected in the present study for its reproducibility, efficiency, and growing use in the fabrication of definitive single-unit restorations (29). Whole-layer polymerization may reduce fabrication time and contribute to consistent production; however, accuracy remains dependent on printing parameters, material characteristics, and post-processing. Previous studies have reported differences in trueness between DLP and stereolithography and have shown that artificial aging may influence the accuracy of printed restorations (30, 31). Direct comparisons using standardized materials, printing orientations, layer thicknesses, and post-curing protocols are therefore needed.

The findings reinforce the importance of fabrication protocols in determining crown surface topography. Nevertheless, the present study measured surface roughness rather than marginal or internal gap, bond strength, cement thickness, or seating accuracy. Consequently, the results should not be interpreted as demonstrating a direct relationship between roughness and adaptation. Previous investigations have shown that fabrication method may influence marginal and internal fit and that both 3D printed and milled restorations can remain within clinically acceptable ranges (32). These observations support further evaluation of the relationship between surface roughness and adaptation but do not establish that one outcome predicts the other.

Digital crown accuracy may also be influenced by scanning, virtual design, alignment, manufacturing parameters, and post-processing. Previous research has indicated that 3D printed zirconia crowns can achieve clinically acceptable trueness, supporting the potential use of additive manufacturing for zirconia-based restorations (33). However, these findings cannot be generalized directly to all printing systems or printable crown materials.

Cement-space settings are another important factor affecting restoration seating and marginal adaptation (34). Adequate cement space may facilitate complete seating and reduce hydraulic pressure during cementation. Although greater internal roughness may increase the potential area for cement interaction, excessive irregularity may produce nonuniform cement layers. Future studies should therefore assess surface roughness together with cement thickness, seating discrepancy, and bond strength.

The thermocycling results also differed among material groups. Milled Zirconia specimens failed during the aging protocol, whereas the 3D printed crowns exhibited resistance to the applied thermal stresses comparable to that of several monolithic ceramic groups. Lava Ultimate crowns had the highest failure rate. Previous clinical investigations have reported relatively low fracture rates for monolithic lithium disilicate and Zirconia restorations (35), and the favorable clinical performance of lithium disilicate has been attributed partly to its ability to achieve durable adhesive bonding to tooth structure (36). Adequate cement space may facilitate complete seating and reduce hydraulic pressure during cementation. Although greater internal roughness may increase the potential area for cement interaction, excessive irregularity may produce nonuniform cement layers. Future studies should therefore assess surface roughness together with cement thickness, seating discrepancy, and bond strength. The thermocycling results also differed among material groups. Milled Zirconia specimens failed during the aging protocol, whereas the 3D printed crowns exhibited resistance to the applied thermal stresses comparable to that of several monolithic ceramic groups. Lava Ultimate crowns had the highest failure rate. Previous clinical investigations have reported relatively low fracture rates for monolithic lithium disilicate and Zirconia restorations (37), and the favorable clinical performance of lithium disilicate has been attributed partly to its ability to achieve durable adhesive bonding to tooth structure (38). Nevertheless, comparisons with clinical studies should be made with caution because the present investigation did not include cyclic mechanical loading.

Possible explanations for the differences in thermocycling survival include variations in elastic modulus, brittleness, thermal expansion behavior, water sorption, adhesive interactions, material microstructure, and post-processing procedures. Zirconia and lithium disilicate may tolerate thermal stresses differently from resin-based materials due to their greater rigidity and structural stability. Resin nanoceramic materials may be more susceptible to water sorption, hydrothermal degradation, or weakening of the adhesive interface. These mechanisms were not directly measured and should therefore be considered hypotheses requiring further investigation.

The present study has several limitations. First, the study was conducted under *in vitro* conditions, which cannot fully reproduce the complex oral environment, including saliva, pH fluctuation, occlusal forces, parafunctional habits, and patient related biological variability. Second, the study was constrained by relatively small and unequal sample sizes across groups, which likely reduced statistical power and limited the generalizability of the findings. The unequal allocation was due to the loss of two specimens assigned to the conventional laboratory-fabricated group during processing at an external laboratory. This loss may have diminished the statistical balance and precision of comparisons involving the conventional laboratory-fabricated crowns. Although unrelated to study outcomes, the smaller sample size in this group may have further compromised balance and precision.

Third, thermocycling was performed without cyclic mechanical loading and therefore represented thermal aging rather than comprehensive functional fatigue. Fourth, the groups differed in material composition, manufacturing techniques, and post-processing requirements, making it difficult to isolate the effect of the fabrication method from that of the material. Fifth, only one DLP printer, one printable ceramic-filled resin, and one cementation protocol were evaluated, which limits the generalizability of the findings to other systems. Sixth, surface roughness was assessed at selected standardized locations rather than across the entire crown surface. Finally, line-profile measurements may be influenced by crown orientation, surface curvature, and the selection of measurement points despite examiner calibration.

Future research should use larger and equally allocated groups and combine thermocycling with cyclic mechanical loading. Studies should compare multiple DLP and stereolithography systems using standardized protocols for printing orientation, layer thickness, post-curing, polishing, and cementation. The effects of cement-space settings, surface treatments, and different resin cements should also be evaluated. Measuring cement thickness, seating discrepancy, bond strength, fracture resistance, and internal and marginal adaptation would help clarify the clinical implications of the observed differences in roughness. Prospective clinical studies are ultimately needed to determine whether these laboratory findings are associated with crown retention, plaque accumulation, periodontal health, marginal discoloration, recurrent caries, or restoration survival.

Overall, internal and marginal surface roughness differed significantly among the evaluated crown materials and fabrication methods. SprintRay Ceramic Crown demonstrated the highest internal roughness, whereas IPS e.max ZirCAD demonstrated the lowest marginal roughness. Because internal and marginal surfaces serve different functions, they should be evaluated separately. The present findings provide laboratory evidence that crown surface characteristics are influenced by the combined effects of material composition, fabrication technique, and post-processing. However, the results should not be interpreted as direct evidence of superior bonding, adaptation, or long-term clinical performance.

## 5 Conclusion

Within the limitations of this *in vitro* study, both material type and fabrication workflow significantly influenced the internal and marginal surface roughness of posterior tooth-colored crowns. Under the evaluated thermocycling conditions, crown retention was more strongly associated with material type than with fabrication method. CAD/CAM, laboratory, and 3D printed crowns exhibited favorable *in vitro* performance following thermal aging. However, the higher failure rate observed with Lava crowns indicates a need for further investigation into the effects of material properties and cementation protocols. As this study assessed thermal aging without cyclic mechanical loading or clinical follow-up, these findings should not be considered direct evidence of long-term clinical performance. Additional laboratory fatigue studies and long-term clinical investigations are required to validate these results.

## Data Availability

The original contributions presented in the study are included in the article/Supplementary Material, further inquiries can be directed to the corresponding author.
